# Identification and Recovery of Valuable Bioactive Compounds from Potato Peels: A Comprehensive Review

**DOI:** 10.3390/antiox10101630

**Published:** 2021-10-16

**Authors:** Beatriz Rodríguez-Martínez, Beatriz Gullón, Remedios Yáñez

**Affiliations:** 1 Department of Chemical Engineering, Faculty of Science, Universidade de Vigo (Campus Ourense), As Lagoas, 32004 Ourense, Spain; beatriz.rodriguez@uvigo.es (B.R.-M.); reme@uvigo.es (R.Y.); 2 Biomedical Research Centre (CINBIO), Universidade de Vigo, 36310 Vigo, Spain

**Keywords:** potato peel, bioactive compounds, multiproduct biorefinery, circular economy

## Abstract

Nowadays, the potato is one of the most cultivated and consumed food crops in the world and, in recent years, its production has experienced a sharp increase. Its industrial processing generates several by-products that are wasted and cause economic and environmental problems. Among them, potato peel stands out, representing up to 10% of the total potato residues obtained in the processing. On the other hand, these wastes, in addition to presenting antioxidant compounds, are rich in interesting chemical compounds of great value in a biorefinery model. This review summarizes the main compounds present in potato skins as well as the most used and innovative extraction methods employed for their isolation, with special emphasis on the fractions with biological activities. In addition, a sustainable biorefinery proposal focused on obtaining high added-value products with potential applications in the pharmaceutical, food, nutraceutical, or cosmetic industries is included.

## 1. Introduction

Currently, food production is greater than its consumption, generating percentages between 30–40% of wasted food which, on many occasions, is dumped in landfills or burned for energy production [1,2]. In this context, fruits and vegetables are the most consumed foods and, due to their high humidity and microbial loads, are highly polluting [2]. Moreover, the industrial processing of fruit and vegetables (in the elaboration of products, such as juices, jams, and dehydrated or frozen products) contributes to increasing by-product production [1]. Due to the current concern about making the food industry more sustainable and the need to reduce the aforementioned environmental problems, there is a great interest in the valorization of these agri-food by-products to obtain added-value compounds [3,4,5], thus supporting the transition towards a circular economy for meeting the “zero waste” goals proposed by the 2030 agenda [6,7].

**Figure 1:**
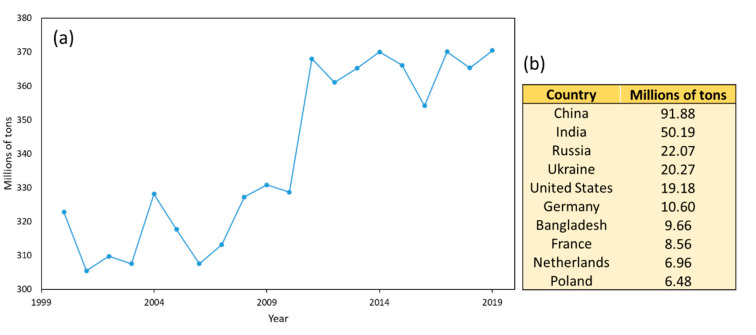
Worldwide production of potatoes from 2000 to 2019 (**a**). Main producers in 2019 (**b**) [11].

The potato, a tuber originally of the Andes [8], is the fourth most important worldwide agricultural product after wheat, rice, and corn [9]. According to data provided by the Food and Agriculture Organization of the United Nations (FAO), in general terms the worldwide potato production has increased in recent years, accounting for 370,436,581 tonnes in 2019 (see Figure 1). Even though more than 100 countries are involved in its production, China and India are the main producers with almost 40% of the total (FAO, 2019). Currently more than 4000 varieties of potato are known, the most widely cultivated being the *Solanum Tuberosum* L. [10].

The potato-processing industry generates large amounts of waste, mainly peel, fried products, screen solids, and wastewater [12]. The potato peel by-product can represent up to 10% of the total potato waste [13], and between 15 and 40% of the fruit, depending on the peeling process selected [14]. This by-product, which lacks value for the feed industry, would be an interesting raw material for the recycling industry, as its processing by environmentally friendly technologies would provide molecules with beneficial properties for human health and multiple industrial applications [9,15].

Potato peel was recently considered a new and important source of dietary fibre as this fraction represents between 40 and 45% of its dry weight (dw) [16]. Concerning its chemical composition, in addition to the high percentage of moisture reported [17], the important contents that should be noted are carbohydrates (63%, where starch accounts for 34%), protein (17%), lignin (10%), ash (9%), and lipids (1%) [13]. Interestingly, this by-product has also been reported as being a source of phenolic compounds that is even higher in the peel than it is in the flesh [18]. Phenolic compounds present well-known biological activities, including those which are antioxidant, antibacterial, antimicrobial, apoptotic, anticarcinogenic, chemopreventive, and anti-inflammatory [2,5]. Therefore, the recovery of bioactive molecules from potato peel is expected to contribute to the development of novel and healthy functional foods [19]. On the other hand, its use as a sustainable feedstock on integrated biorefineries could open up a wide spectrum of applications [15].

**Figure 2:**
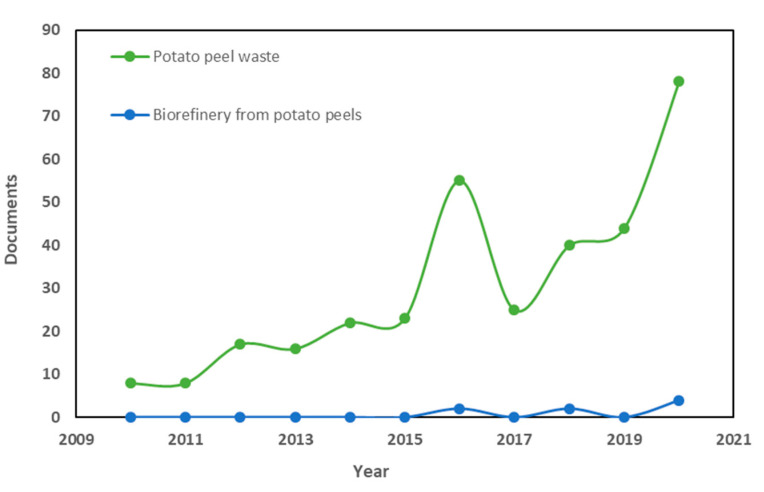
Research tendencies in “potato peel waste” and “biorefinery from potato peels” from 2010 to 2020. Source Scopus (3 June 2021).

The overall increase in publications observed in the potato peel field over the last five years could have been boosted by the relevance of its bioactive compounds. As can be seen in Figure 2, introducing the search term “potato peel waste” in Elsevier′s SCOPUS bibliographic database, between 2010 and 2015 only 94 articles were published compared to the period of 2016–2020, in which the number rose to 244. Among the most used key words in these publications are “biomass”, “agricultural wastes”, or “waste management”. When the search “biorefinery from potato peels” was carried out, it was confirmed that it is a novel topic as only a few articles have been published since 2015 (see also Figure 2).

Based on the aforementioned investigation, future research aimed at developing effective technology for the integral valorization of these by-products would be necessary, both for the extraction of bioactives and for the production of other valuable products. Therefore, this review summarizes the main studies focused on the recovery of the interesting compounds present in potatoes and the different extraction technologies employed; also included is a green biorefinery model based on multi-product processes in cascade for these by-products.

## 2. Main Components of Potato Peel and Their Bioactivities

Potato peel is considered an interesting source of several bioactive compounds, among which are phenolic compounds, glycoalkaloids, polysaccharides, proteins, and vitamins. These compounds will be described in the following sections.

### 2.1. Phenolic Compounds

Phenolic compounds are secondary plant metabolites observed in different species, including those of the Solanaceae family. These compounds are responsible for functions such as UV protection, pigmentation, disease resistance, and the defense of plants against invading pathogens [20,21]. The antioxidant activity of the phenolic compounds gives them important applications in the food field as these compounds are the most abundant antioxidants in the human diet [22].

**Figure 3:**
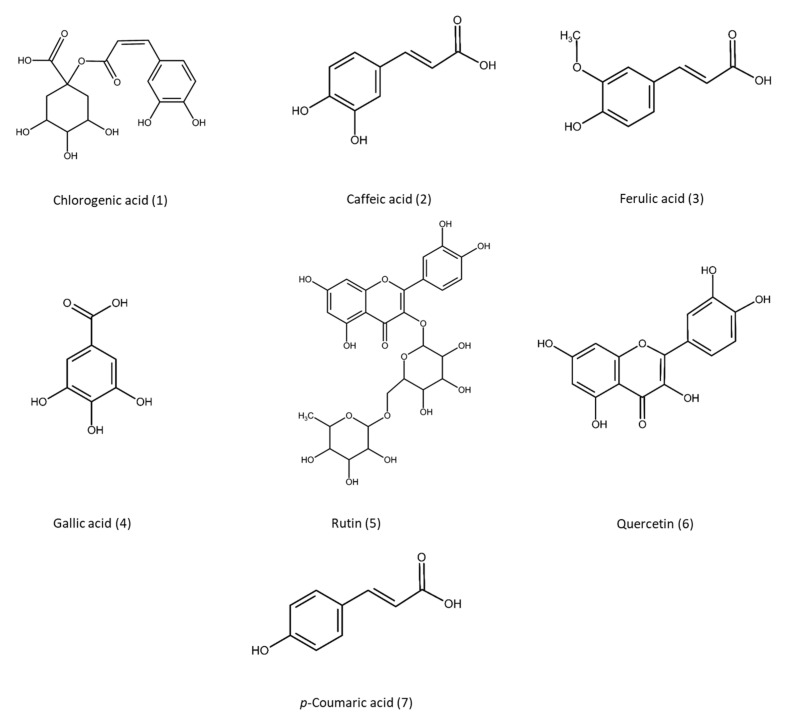
Chemical structure of some phenolic compounds identified in potato peel [23,26,31].

In the case of potatoes, about 50% of their phenolic compounds were found in the peel and adjoining tissues while this percentage decreased towards the potato center [21]. Table 1 summarizes the total phenolic content and the main phenolic compounds detected in several potato peel varieties as well as the studied bioactivities. In order to provide additional information about the main biomolecules identified in this by-product, their chemical structures were included in Figure 3. In the potato peel, phenolic compounds can be found in free, soluble (esterified), or insoluble bound form. Although most studies only focus on the free phenolic compounds, the great contribution of the esterified and bound phenols to the total phenolic compounds heightens their importance [23]. As can be seen in Table 1, phenolic acids are the main phenolic compounds present in the potato peel, representing around 3.43% of the aqueous extract [24,25]. They include hydroxycinnamic acid derivatives, such as chlorogenic acid, caffeic acid, p-coumaric acid, ferulic acid, and hydroxybenzoic acid derivatives, such as vanillic acid, protocatechuic acid, gallic acid and p-hydroxybenzoic [23,26]. Among them, the predominant one is chlorogenic acid (49–61% of total phenols), followed by caffeic (2.3–19.9%), gallic (7.8%), and protocatechuic (0.21%) acids [27,28]. The wide range reported for these components (see Table 1) could be due to the influence of storage temperature or exposure to light that causes the transformation of chlorogenic acid into caffeic acid and quinic acid [27]. It should be noted that vanillic and ferulic acids, among others, were found on the skin in higher concentrations than in the flesh [29]. Flavonoids, the most common group of phenols in plants, are responsible for the flavour and colour. They were identified as the second family of phenolic compounds in potato peel, being mainly flavonols and anthocyanins [26,30]. Anthocyanins are glycosidic water soluble pigments especially present in red and purple potato varieties [26,29]. Moreover, in previous works, the presence of catechin [25], kaempferol, and rutin were also detected in potatoes [30], as well as quercetin in potato peel [31].

The high content of anthocyanins observed in some genotypes of red or purple colour, such as the Siècle or Purple Majesty varieties, demonstrate the great influence of the variety on this parameter. As can be seen in Table 1, while the yellow varieties (Russet, Innovator, and Yellow) only present a total anthocyanins content of between 0.002–0.004 mg/g, the “Purple” variety reaches values of 0.068 mg/g [23]. Therefore, the selection of the genotype could be made as a function of the future application [33].

The determination of the total phenolic compounds in potato peel extracts is commonly conducted by the Folin-Ciocalteu method. However, for the identification and quantification of individual phenolic compounds present in potato peel extracts, techniques such as High-Performance Liquid Chromatography (HPLC) [17,19,32], using different detectors, such as the DAD (Diode Array Detector) [23,31,34] and the ESI-MS (Electrospray Ionization and Mass Spectrometer) [28] are widely applied due to their high efficiency. For instance, Riciputi et al. [28] carried out the identification and quantification of phenolic compounds in ethanolic extracts obtained by the ultrasonic treatment of five potato by-products using HPLC-DAD-ESI-MS. The results showed the presence of 12 compounds, among which were chlorogenic acid, caffeoylquinic isomers, and feruloyl derivatives.

Albishi et al. [23] determined by HPLC-DAD that the most abundant free phenolic acid in the Innovator, Russet, and Purple peels was the chlorogenic acid, while in the Yellow potato it was the caffeic acid. Another option would be the Ultra-Performance Liquid Chromatography (UPLC), combined with Photodiode Array Detection (PDA) and coupled with an Ultra-High Resolution Time of Flight Mass Spectrometer (UHR-TOF-MS) [29]. Oertel et al. [29] conducted a study dealing with the phytochemical characterization of 19 potato by-product samples using UPLC. The results obtained showed differences in the anthocyanin profiles of the potato peels, which strongly depend on the genotype.

Phenolic compounds are well known for their health-promoting biological activities as they possess antimutagenic, antimicrobial, anticancer, antiglycemic, and antioxidant properties [21,32]. As can also be seen in Table 1, potato peel studies are mainly focused on the antioxidant activity determination. A positive correlation between the total phenolic compounds and the antioxidant activity has previously been reported in potato extracts and can be expressed through Pearson′s correlation coefficient [19,28]. In particular, the presence of caffeic and chlorogenic acids in the extracts was associated with high antioxidant activities [18]. Therefore, chlorogenic acid and their derivatives have recently been gaining attention in the nutrition field because of their preventing or controlling of the formation of free radicals. In addition, it has been reported that chlorogenic acid could modulate lipid metabolism and glucose, thus it could improve many disorders, such as hepatic steatosis, cardiovascular disease, and diabetes, as well as obesity. It has also been suggested that chlorogenic acid plays an important role as an antihypertensive agent [35]. With respect to caffeic acid, several in vitro and in vivo studies have highlighted its anti-hepatocarcinoma activity [36]. Flavonoids, such as rutin, also found in potato peel, exhibit antimicrobial, antifungal, and antiallergic activities. In addition, the flavonoids also present pharmacological benefits for the treatment of various chronic diseases, such as cancer, diabetes, hypertension, and hypercholesterolemia [37]. The major anthocyanins content in the purple and red potato varieties provide them with higher antioxidant, anticarcinogenic, and anti-inflammatory activities. Moreover, anthocyanins, recognized as potent dietary antioxidants, help to prevent and treat diabetes and heart conditions [23,29]. Regarding antimicrobial activity, it was observed that potato extracts reduce the number of harmful bacteria in the intestinal tracts of pigs [38] and act against some human pathogens, such as *Escherichia Coli* and *Salmonella Typhimurium* [24]. Finally, to highlight the effectiveness of natural potato peel, aqueous extracts can avoid the oxidation of sunflower oil in comparison with a synthetic antioxidant such as butylated hydroxyanisole (BHA) [27].

### 2.2. Glycoalkaloids

Steroidal glycoalkaloids (SGAs) are secondary metabolites that are normally found naturally in different organs of plants, mainly in the solanaceous species [39,40,41,42]. They are made up of a hydrophobic C27-steroidal base, the aglycone, with several sugar groups attached at the C3-hydroxyl position [39,43]. Table 2 summarizes the total content of glycoalkaloids and the main glycoalkaloids compounds identified and quantified in several potato peel varieties as well as the methods employed. As given in Table 2, among the most important are the triose glycosides of solanidine [39,40,42,43,44,45], being normally the most abundant α-chaconine, followed by α-solanine [43,44,46,47,48]. Other glycoalkaloids found in lower concentrations (see Table 2) were the solanidine (an alkaloidal aglycone), the demissidine (its dehydrogenated form), α-tomatine, and commersonine [41,45]. The highest concentration of glycoalkaloids was observed in sites with high metabolic activity, such as the potato skin and the adjacent tissues (up to 1.5 mm thick), as well as in the eyes and the damaged areas [47].

Glycoalkaloids are compounds characterized by their bitter taste and toxicity, comparable to arsenic [42,46]. Their ingestion in high concentrations (greater than 3–5 mg/kg of body weight) is associated with detrimental effects on human health, causing colicky pain in the abdomen and stomach, diarrhea, gastroenteritis, vomiting, confusion, hallucinations, rapid pulse, fever, and even neurological disorders [41,42,44,45,47,49]. Therefore, the concentration of glycoalkaloids in the potato’s raw material must be less than 20 mg/100 g fresh weight [45,46]. Furthermore, because of the higher α-chaconine toxicity, it is usually considered as ratios of α-solanine/α-chaconine close to two [47]. However, SGA plays an important role in the defense of the plants against pests, pathogens, and insects [39,41,42,43,44]. Hence, the rising interest in the study of glycoalkaloids as a source of permanent disease resistance, employing potatoes grown by introgression [39,40].

There are various cultural, genetic, and storage factors that affect the concentration of glycoalkaloids in potato peel [46]. Concerning the variety, it was shown that the blue-fleshed variety showed the major concentration (5.68 mg/100 g of fresh weight), followed by the red-fleshed (5.26 mg/100 g of fresh weight), and the yellow or cream-coloured flesh [47]. On the other hand, a concentration increase may be caused by several environmental stresses, such as exposure to light during storage, high temperatures during the growing season, soil fertility, frost damage, premature harvest, mechanical damage, etc., [40,42,43,45,50] while a decrease is experienced in cooked and processed products [46].

Despite their toxicity, glycoalkaloids are interesting due to their bioactivity as both SGA (α-chaconine and α-solanine) and the aglycone alkaloids have anti-inflammatory and anti-cancer properties. However, synthetic modifications should be explored to enhance their bioactivities and reduce their toxicity for further phytopharmaceutical industrial applications [41]. Recent findings show that aglycone alkaloids have greater anti-inflammatory activities while steroidal glycoalkaloids principally exhibit anticancer properties [41,44]. These last compounds have also been used in the treatment of the Herpes simplex virus [40,51]. On the other hand, glycoalkaloids act on the cholesterol content of membrane cells. Thus, compounds such as α-chaconine and α-tomatine form strong complexes with cholesterol, which interact with the cell membrane and cause its rupture [52]. Glycoalkaloids have also gained interest as possible precursors for the production of hormones and antibiotics, and for use in certain skin diseases [34]. Finally, it should be noted that among the two main glycoalkaloids, α-chaconine presents bioactivities five times higher than those of α-solanine [48,52].

### 2.3. Polysaccharides

Polysaccharides are macromolecular substances that are found naturally in animals, plants, and microorganisms [53]. They are constituted by different monosaccharides linked by α or β glycosidic bonds [54] and are considered a natural source of bioactive compounds [55]. Thanks to their diverse structures and functional properties, polysaccharides can have commercial applications, such as texture improvement, water retention, and emulsion stabilization [17]. In particular, starch and non-starch polysaccharides (pectin, cellulose, and hemicelluloses) were found in potato skins [13] in percentages of 46% and 26.6% of the dry matter, respectively [56]. The monosaccharide composition in potato peel polysaccharides shows a high percentage of glucose (76.25%), followed by galactose (3.84%), rhamnose (0.506%), and arabinose (0.19%) [54]. With these percentages, it can be concluded that glucose is the backbone of the polysaccharides, while galactose, rhamnose, and arabinose are found in the branched structure [57]. In a recent study, high percentages of water-soluble polysaccharides were isolated from potato peel, obtaining concentrates with purities around 95% [58].

Starch is a biomolecule formed by two types of glucose polysaccharides, amylose (slightly branched) and amylopectin (highly branched). Due to its properties, such as thermoplastic capacity and film-forming ability and its natural availability in different plants, starch can be used in the production of bioplastics as well as in the paper, textile, and food industries [55,59].

Pectin is a natural polysaccharide consisting mainly of D-galacturonic acid linked by α-(1-4) glycosidic [60]. A recent study reported that potato waste pectin contains an appreciable amount of rhamnogalacturonan I (RG-I), a hairy region of pectin [61], made up of a backbone of α-d-galacturonic acid and α-l-rhamnosyl residues and branched side chains of galactose, arabinose, and rhamnose [62]. Pectin has been used for years in industry as a thickening and gelling agent and colloidal stabilizer [60]. In particular, potato pectin is highly acetylated and shows a low degree of esterification. These facts promote their gelling properties and make them better candidates for different applications than other pectin [56,62].

The polysaccharides extracted from the skin of potatoes, like other plant polysaccharides, have a high antioxidant activity as they capture free radicals in the diet to prevent oxidative damage [57]. These compounds can also present biological activities, such as those which are antitumor, antiviral, immunostimulant, anti-inflammatory, and anticoagulant [17,63]. In this context, it should be noted that the good radical scavenging activity exhibited by potato peel polysaccharides might be due to a possible fraction of the esterified phenolic compounds that may have precipitated with them [17]. Among the different polysaccharides, potato peel β-glucans stand out as they can be effective in treatments for cancer and infections because they are recognized as non-self-molecules in the immune system. These compounds can act against leukaemia, microbial infections, hypercholesterolemia, or diabetes. A recent study reported improved technological and nutritional characteristics for oligosaccharides. They exhibit potent biological activities acting as anticancer drugs and for these reasons the therapeutic and food fields are giving them great importance as substitutes for polysaccharides [57].

### 2.4. Protein and Amino Acids

Amino acids are compounds that combine to form proteins. The human body cannot synthesize exogenous amino acids, but they are necessary so they must be acquired through food intake. Potato peel is considered a great source of protein with a well-balanced amino acid composition, which makes it a raw material of interest [64]. Potato skin has a higher nitrogen and amino acid content than the flesh [65]. Although the percentage of protein in potatoes is relatively low (0.7–4.6%), it may contribute to the protein intake due to the large amount consumed. Potato protein contains 18 amino acids, including the essential 9: tryptophan, leucine, isoleucine, valine, threonine, lysine, methionine, phenylalanine, and histidine [66]. In a previous study, it was observed that they represent around 25% of the total amino acid, with valine being the one with the highest presence (with 5.3% of the total amino acid), followed by lysine (4.5%) and leucine (4.3%). Moreover, the amino acid found in the highest concentration was aspartic acid (23.3% of the total amino acid of protein), followed by glutamic acid (15.4%) and arginine (6.5%) [67].

There are different human and natural factors that could affect the content and composition of the amino acids present in potatoes. Knowing this content is important as amino acids indirectly affect the taste (due to aspects such as the ratio of amino acids to sugars or starch content) and the aromatic properties of potatoes [66]. Among human factors, it was shown that the exogenous amino acid content was higher when the nitrogen fertilization level was lower [64]. Regarding natural factors, it was observed that growing conditions affect the amount of amino acid. Higher contents were reported in the flowering phase and in spring. During storage, the deterioration in the flavour of the potato is associated with an increase in the amount of amino acid and a decrease in the starch content [66].

The free amino acids coming from potato peel play a dual role in the human diet. These compounds can react with free sugars, producing browning products, such as acrylamide, well-known as a harmful component for cells, animals, and probably also humans. However, they are major contributors to their nutritional and protein values, as well as correlating with cardiac function. On the other hand, the non-protein amino acids and the amino acid metabolites present in potato skin are very beneficial due to their bioactivities. For instance, β-alanine improves exercise performance and fatigue in humans; α-aminoadipic acid is a diabetes risk marker and a potential modulator of glucose homeostasis in humans; 4-aminobutyric acid decreases psychic anxiety; β-aminoisobutyric acid prevents obesity and protects from metabolic disease and cardiometabolic risk factors; L-Carnitine prevents adverse effects on the heart; hydroxylysine strengthens the mechanical properties of animal and human collagen tissues and artificially created neotissues; L-ornithine relieves stress and improves sleep quality; and phosphoserine has the potential to reduce Alzheimer′s disease and to enhance cell-based construction in bone tissue engineering [68].

### 2.5. Vitamins and Minerals

Vitamins are essential nutrients that the body cannot synthesize, thus they must be provided by daily diet [64]. Moreover, minerals play a crucial role in several metabolite processes [69]. The potato is an important source of potassium (K), phosphorus (P), iron (Fe), sodium (Na), magnesium (Mg), and vitamins C, B1, B6, and B9 [64,70]. The relevance of the influence of fertilizers in potato cultivars to the antioxidant activity of vitamins has been studied, associating a descent in the content of vitamin C with the supply of nitrogen and potassium to the plants [64].

## 3. Extraction Technologies for Bioactive Compound Recovery from Potato Peel

Different conventional and non-conventional extraction methods have been studied for the recovery of high-interest compounds from potato peel [71]. An extraction is considered effective when maximizing the isolation of the target compounds while presenting minimal degradation. Table 3 summarizes the extraction conditions, the phenolic components, and the bioactivities found for various potato varieties using different technologies. As can be seen in Table 3, several parameters can affect the extraction yield as well as the quality and composition of the resulting extracts. Another relevant aspect in this field to consider is the employing of environmentally friendly technologies and solvents [72].

### 3.1. Conventional Methods

Conventional solid–liquid extraction methods such as Soxhlet or heat reflux are commonly used for the recovery of bioactive compounds from potato peel [16,26]. The extraction efficiency depends on various factors, such as the type of solvent, the solvent-to-material ratio, the extraction method, the pressure, the time, and the temperature and particle size [41,76,77]. The choice of the extraction solvent is considered the most significant parameter, with certain organic solvents, such as ethanol, methanol, acetone, or diethyl ether, in addition to aqueous alcohol mixtures [26,76,77], being among the most employed. It is difficult to know which ones report the best extraction yields in plant matrices, due to their different chemical characteristics [76]. Concerning potato peel, when analysing the data collected in Table 3 for the conventional methods, the highest total phenol was obtained using acetone (650 mg GAE/100 g db) [32].

These extraction techniques are not environmentally friendly because they use large amounts of toxic solvents that are not considered safe for food applications [41]. Moreover, sometimes a completely dry material is required and there is a risk of the degradation of the heat-labile constituents, due to high temperatures for prolonged times, and the yield and selectivity are lower compared to those of other more innovative methods [26,63]. For all these reasons, green solvents are proposed as an interesting alternative to reducing the environmental impact by conventional techniques. The main requirement for a solvent to be considered green is that it must be environmentally friendly throughout its life cycle. In this context, natural deep eutectic solvents (NADES) stand out as they are formulated by a mixture of two or more natural components, such as non-toxic quaternary ammonium salts and uncharged hydrogen-bond donors, that interact through intramolecular hydrogen bonds. NADES offer different advantages, including low cost, easy preparation from readily available materials, negligible volatility, and low toxicity [78].

### 3.2. Emerging Extraction Methods

Due to the drawbacks of the conventional extraction methods, as can be seen in Table 3, other novel alternatives for the isolation of bioactive compounds from potato skins have been proposed [73]. Among these techniques are: Ultrasound-Assisted Extraction (UAE), Microwave-Assisted Extraction (MAE), Pressurized Liquid Extraction (PLE), Supercritical Fluid Extraction (SFE), and Supercritical CO_2_ Extraction (SSCO_2_).

#### 3.2.1. Ultrasound-Assisted Extraction

UAE or sonication is a simple and green extraction technique for bioactive compounds [26]. This method is based on the use of cavitation bubbles near the surface of the plant raw material, thus causing an increase in pressure and temperature which deteriorates the plant cell walls [79]. Moreover, sonication allows a greater penetration of solvent, increasing the contact surface between the solid and liquid phase, improving solute diffusion and therefore resulting in a higher extraction yield [26]. This technology has been used for the efficient extraction of phenolic compounds [73], pectin [79], and amino acids [71].

Among the main advantages of UAE, compared to other conventional methods, are the increase in the recovery yields, the reduction in solvent consumption and treatment time, and a greater repeatability. In addition, UAE is a technology which is versatile, clean, and easy to use and to scale up for commercial use; it is environmental friendly and cost-effective [44,73]. The application of UAE allows the obtaining of purer thermosensitive bioactive compounds at a lower temperature, thus avoiding their degradation [26]. However, this technology could degrade some organic compounds [44].

Analysing the data summarized in Table 3, it is observed that, as expected according to the previously described advantages, UAE resulted in higher total phenols recoveries in the different potato varieties. For example, for the ratona morada variety, UAE yielded 1770 mg GAE/100 g db, while conventional extraction yielded only 650 mg GAE/100 g db [32], whereas values of 7.67 mg GAE/g db and 3.28 mg GAE/g db were obtained for the Lady Rosetta variety [73].

UAE is classified as indirect ultrasound-assisted extraction (IUAE) and direct ultrasound-assisted extraction (DUAE), depending on if an ultrasound bath or a probe is used, respectively. DUAE presents a higher level of ultrasonic radiation, which accelerates the extraction of bioactives from potato peel, being able to be up to five times faster than IUAE at short reaction times. However, as can be seen in Table 3, both methods reached similar maximum total phenolic yields at 30 min (9.33 mg GAE/g db for DUAE and 9.09 mg GAE/g db for IUAE) [74].

#### 3.2.2. Microwave-Assisted Extraction

Microwave energy is non-ionizing radiation that can penetrate materials without modifying the chemical structure of the key components [79]. The direct effect of microwaves on molecules by ionic conduction and dipole rotation causes an increase in temperature [26], encouraging the bioactive compounds to dissolve in the solvent due to the increase in pressure generated by the water vapour on the cell walls [79].

One of the advantages of this technique is that it is a clean and a fast method that presents high extraction yields. It is also selective, easily controllable, energy-saving, and uses small amounts of solvents compared to conventional methods, making it a highly cost-effective solution [26,80]. However, it has the disadvantage of degrading several heat-labile organic compounds [44].

Despite the fact that MAE has been widely reported for the extraction of bioactive compounds from different agro-industrial wastes, its application to the by-products of industrial potato processing is still scarce. As can be seen in Table 3, only one research work has been focused on the recovery of phenolic antioxidants from potato peel with this technology [75]. In this study, the authors optimized the microwave extraction conditions (extraction time, solvent concentration, and microwave power) through Response Surface Methodology (RSM) to extract antioxidants from these by-products. Under optimal conditions (67.33% aqueous methanol, 15 min, and 14.67% power), MAE resulted in a total phenolic content of 3.94 mg/g db. However, the conditions that led to increased antioxidant activity using the DPPH assay (74% of inhibition) were 100% methanol concentration, 5 min of extraction time, and 10% of level power. It is necessary to carry out more research work focused on the use of this technology in order to assess the suitability of MAE in recovering different biomolecules from potato by-products.

#### 3.2.3. Supercritical Fluid Extraction

SFE is an efficient and environmentally friendly extraction technique used for the selective isolation of components of interest. Although it has similar advantages to those observed in UAE, it is still not profitable due to its high operating and capital costs [44,81]. In this technology, the supercritical fluid used as a solvent adjusts the solubility through physical parameters, such as the temperature and pressure, thus causing the extraction of analytes of different polarities and molar mass. In addition, it reduces the volume of the solvents used during the extraction [82].

The supercritical fluid most commonly used in this type of extraction is CO_2_, although other solvents, such as nitric oxide, ethane, propane, n-pentane, ammonia, and water have also been tested [82]. However, CO_2_ has some drawbacks such as its low polarity, which can limit the efficient extraction of many organic compounds from agricultural residues. Moreover, SFE requires dry raw materials and organic co-solvents to increase the extraction efficiency [63].

To the best of our knowledge, there is no study on the extraction of bioactive compounds from potato peel using this technique. However, SFE has been successfully applied to recover valuable compounds from various food wastes [83,84].

#### 3.2.4. Pressurized Liquid Extraction

PLE is based on the use of high pressures to maintain the solvent, usually water, at high temperatures (between 100 and 374 °C) [26]. The high pressure and temperature employed promote the solubility of the key bioactive compounds, the diffusion rate of the solvent, and the mass transfer as they decrease the viscosity and surface tension of the reaction media [41].

PLE has some advantages over conventional methods, including shorter extraction times, higher selectivity and lower consumption of toxic organic solvents [26]. In addition, PLE is an automated process that can take samples in environments without oxygen or light, but it has not yet been scaled up to an industrial level as complex and expensive equipment would be required [26,41].

As with UAE and MAE, there are several factors which also influence the extraction efficiency of the PLE processes. In this respect, Wijngaard et al. [34] evaluated the operating variables of PLE in order to optimize the recovery of polyphenols from industrially generated potato peel. This study examined the impact of the ethanol concentration (42–98%) and the temperature (65–135 °C) on antioxidant activity, phenol level, and caffeic acid concentration using a Central Composite Design. The optimal conditions for the isolation of the desired compounds were 70% ethanol and 125 °C. In addition, the authors also compared this technology with the conventional extraction method using 100% methanol as a solvent. In this case, PLE led to better results: DPPH of 339 vs. 200 mg TE/100 g db; phenolic contents of 368 vs. 126 mg GAE/100 g db; and caffeic acid contents of 536 vs. 465 g caffeic acid/g db. Moreover, Hossain et al. [41] optimized the steroidal alkaloids extraction by response surface methodology. Under optimal operational conditions (80 °C and 89% methanol), the maximum concentrations of glycoalkaloids were 597, 873, 374, and 75 μg/g db for α-solanine, α-chaconine, solanidine, and demissidine, respectively. In addition, a higher yield of glycoalkaloids was obtained using PLE compared to the conventional extraction (1.92 mg/g db vs. 0.981 mg/g db).

#### 3.2.5. Sequential Hydrothermal Extraction

An alternative to the single-stage processes is the SeqHTE, based on the extraction of several bioactive molecules according to their water affinity at different temperatures. In a two-stage SeqHTE process, the lower viscosity and interfacial surface tension of subcritical water facilitate the penetration of the solvents and the mass transfer [17]. The first pretreatment stage is carried out at low temperatures where solid–solute interactions are broken, biomass porosity is increased, and polar interactions between the water and the target molecules are slightly favored. In the second stage, the subcritical water at high temperatures promotes low polar interactions and causes more significant thermal and mass transfer effects [17,63].

Among the advantages of SeqHTE technology, it has been that found that the sequential processes reduce the amount of spent solid waste and prevent the excessive thermal degradation. In addition, the flexibility of the methodology opens its applicability to different feedstocks, allowing the recovery of selective target compounds [17,63].

Recently, Martínez-Fernández, Gu, et al. [63] evaluated the potential of SeqHTE for recovering biomolecules from potato peel. Using an extract mass flow of 4605.27 kg/h in the first stage and 5768.54 kg/h in the second stage, polyphenol flows of 17.01 kg/h and 3.72 kg/h were obtained, respectively.

## 4. A Biorefinery Strategy for Integral Use of Potato Peel

The use of agro-industrial residues as feedstock in an integrated biorefinery to get several high added-value products will allow solving environmental problems related to the inadequate management of these by-products, while contributing to a better economic performance of the food sector. In addition, the re-incorporation of these by-products into other productive processes is key to promoting the implementation of a bio-economy based on “zero waste” [7].

**Figure 4:**
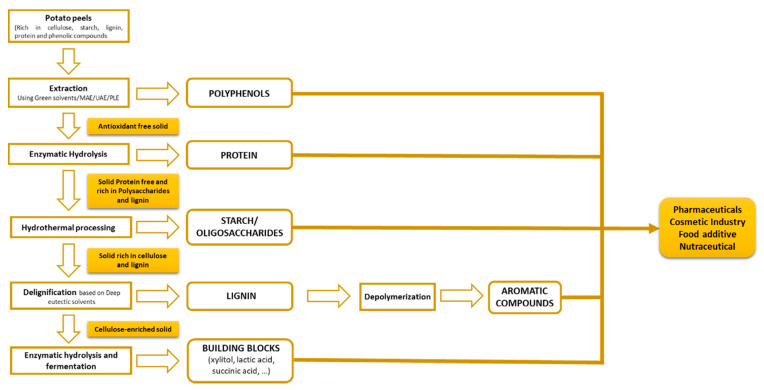
Integrated biorefinery model for potato peel waste.

According to the chemical composition found in the literature for potato peel (cellulose, starch, lignin, protein, phenolic compounds, etc.), several processing routes for their optimal valorization could be evaluated. In this context, Figure 4 shows a possible biorefinery scheme based on multiproduct cascade processes using by-products from the potato processing industry.

The first stage would lead to the recovery of phenolic compounds using green solvents and innovative extraction methods, such as UAE, MAE, or PLE. These technologies combined with emerging solvents (as the deep eutectic solvents) have been widely applied for the isolation of various biomolecules, including polyphenols, polysaccharides, pectin, and protein, inter alia, from the multiple by-products generated in the agri-food sector [5]. The antioxidant-free solid could be subjected to enzymatic hydrolysis for protein recovery. Moreover, several authors have also proposed the use of hybrid processes based on the application of UAE or MAE coupled with enzymatic extraction to improve protein extraction efficiency [85,86].

After this second processing step, the extraction of starch or oligosaccharides from the previously deproteinized solid could be performed via hydrothermal treatment. This technology has been successfully applied for the solubilisation of different polysaccharides present in agri-food residues, including peanut shells [87], melon peels [88], chestnut shells [89], and vine shoots [90], among others.

A delignification stage (employing deep eutectic solvents (DES)) of the spent solid recovered from hydrothermal processing would solubilize lignin, leading to a cellulose-rich solid fraction. The application of DES as solvents to dissolve lignin has recently been evaluated in the scientific literature. In this context, Rico et al. [88] and Del Castillo-Llamosas et al. [7] also proposed biorefinery strategies based on DES for the selective solubilisation of cellulose and lignin from melon and avocado peels, respectively.

The solid rich in cellulose could be valorized through biotechnological processes for the production of different high-value bioproducts, such as lactic acid, succinic acid, or xylitol. On the other hand, different aromatic building blocks could be obtained via the depolymerisation of the lignin fraction.

The biorefinery model presented in this review is an appropriate strategy for the management of waste from the potato processing industries. In addition, the proposed treatment stages based on the use of green solvent chemicals and sustainable innovative technologies will contribute to cutting down the environmental impact through the whole production process.

## 5. Conclusions and Future Prospects

Potatoes are one of the most widely used agri-food products in the human diet around the world. However, their processing causes the generation of large amounts of by-products, such as peel, provoking important environmental and economic problems if not managed properly. Based on the information compiled in this review, we can conclude that the potato peel can be used to obtain bioactive compounds with high added-value, including polyphenols (phenolic acids and flavonoids); glycoalkaloids (α-chaconine and α -solanine, among others); polysaccharides (starch and pectin, among others); etc. Therefore, the recovery of these bioactive compounds not only mitigates environmental problems but also enhances the profitability of the food industry. To achieve this objective, it is necessary to apply innovative technologies that would allow the obtaining of higher extraction yields, while maintaining the properties of the obtained extracts. In this framework, the implementation of a biorefinery using potato skins as the main source could open future industrial applications that would allow the full use of this by-product.

## Figures and Tables

**Table 1:** Total phenolic content and the main phenolic compounds detected in different potato peel varieties.

Potato Variety	Concentration (mg/g db)									Antioxidant Assay					Ref
	1	2	3	4	5	6	7	8	9	I	II	III	IV	V	
Siècle	2.79	0.52	-	-	-	-	-	-	-	1.07	0.55	90	-	-	[19]
Purple majesty	2.19	0.72	-	-	-	-	-	-	-	0.72	0.50	88	-	-	
Dakota pearl	0.78	0.47	-	-	-	-	-	-	-	0.65	0.38	87	-	-	
FL 1533	1.33	0.44	-	-	-	-	-	-	-	0.50	0.35	85	-	-	
Vivaldi	1.54	0.26	-	-	-	-	-	-	-	0.50	0.35	85	-	-	
Yukon gold	0.86	0.41	-	-	-	-	-	-	-	0.43	0.28	82	-	-	
Ratona morada	0.38	-	-	-	-	-	-	-	1.77 *	-	-	-	39.81 **	0.410	[32]
Curiquinga	0.44	-	-	-	-	-	-	-	1.91 *	-	-	-	49.98 **	0.412	
Russet Burbank	-	-	-	-	-	-	-	-	7.0	-	-	-	21–42 stage 1 33–65 stage 2	-	[17]
Mixed peels	-	-	-	-	-	-	-	-	8.0	-	-	-	15–34 stage 1 21–42 stage 2	-	
Bintje	1.97	0.24	0.06	-	-	-	-	-	3.23	-	-	-	28.25 ***	4059.05 ^3^	[28]
Challenger	1.27	0.22	0.05	-	-	-	-	-	2.48	-	-	-	21.04 ***	3179.92 ^3^	
Daisy	4.10	0.16	0.12	-	-	-	-	-	7.23	-	-	-	42.30 ***	5745.18 ^3^	
Innovator	2.52	0.30	0.06	-	-	-	-	-	5.04	-	-	-	39.88 ***	5138.86 ^3^	
Fontane	3.04	1.22	0.12	-	-	-	-	-	6.15	-	-	-	46.40 ***	6037.12 ^3^	
Fianna	3.46	3.33	0.03	2.33	0.05	0.11	-	-	-	-	-	-	0.38 ****	1.4 ****	[31]
Innovator	1.29	1.09	0.85	-	-	-	5.30	0.002	11.5 *	1907.26 ***	1.86	-	32.75 ***	-	[23]
Russet	1.35	0.99	0.57	-	-	-	0.09	0.004	9.32 *	1533.88 ***	1.10	-	28.01 ***	-	
Yellow	0.17	0.30	0.13	-	-	-	0.03	0.003	4.54 *	560.93 ***	0.70	-	13.88 ***	-	
Purple	3.65	0.92	0.07	-	-	-	0.07	0.068	13.9 *	1179.76 ***	2.14	-	17.10 ***	-	

GAE: Gallic acid equivalent, db: dry biomass, fdb: freeze dry biomass, FRSA: Free Radical Scavenging Activity, TEAC: Trolox equivalent antioxidant capacity, FRAP: Ferric Reducing Antioxidant Power, ABTS: 2,2′-azino-bis-3-ethylbenzothiazoline-6-sulfonic acid, DPPH: α,α-diphenyl-1-picryl-hydrazyl radical scavenging, AE: Antiradical efficiency; * (mg GAE/100 g db), ** (AE × 10^−3^), *** (µmol TE/g db), **** (mmol TE/g); 1. Chlorogenic acid, 2. Caffeic acid, 3. Ferulic acid derivative, 4. Gallic acid, 5. Rutin, 6. Quercetin, 7. p-Coumaric acid, 8. Total anthocyanin, 9. TPC; I. FRAP (mg GAE/g db), II. TEAC (mM/g db), III. FRSA (%), IV. DPPH (%), V. ABTS (mM TE/g bs).

**Table 2:** Total glycoalkaloid content and the main glycoalkaloids detected in different potato peel varieties.

Potato Variety	Concentration (mg/100 g db)			Identification Method	Ref
	α-Solanine	α-Chaconine	Total SGA		
n.d.	429	328	1012	UPLC-MS/MS	[41]
Desireé	5294 *	11,881 *	17,175 *	UPLC–Triple Quadrupole–MS	[43]
n.d.	1.6	15.40	-	UHPLC-qTOF-MS	[44]
Conventional Gold	25.3	67	92	HPLC	[48]
Conventional Red	41.2	12.97	17.09		
Conventional Russet	21.5	42.4	63.9		
Organic Gold	75	28.3	35.8		
Organic Red	23.9	61	85		
Organic Russet	37.4	11.80	15.50		
*S. acaule* ssp. *acaule*	traces	traces	121.05	LC-ESI-MS and HPLC-DAD	[45]
*S. ajanhuiri*	traces	25.86	1449.17		
*S. alandiae*	918.17	1743.17	2664.57		
*S. bulbocastanum* ssp. *bulbocastanum*	10.66	34.93	45.59		
*S. chaucha*	61.82	116.93	829.47		
*S. chomatophilum*	2.99	16.98	64.07		
*S. curtilobum*	37.68	106.82	144.50		
*S. demissum*	traces	9.13	889.58		
*S. maglia*	13.13	31.51	227.34		
*S. microdontum*	33.68	50.01	1870.31		
*S. pascoense*	42.83	15.26	822.41		
*S. phureja* ssp. *Phureja*	425.61	974.70	1453.26		
*S. polyadenium*	6.14	47.06	53.20		
*S. raphanifolium*	1.68	27.19	28.87		
*S. sparsipilum*	563.86	948.10	1520.31		
*S. tarijense*	traces	46.77	2369.52		
*S. tuberosum* ssp. *andigena (white)*	208.63	52.45	355.79		
*S. tuberosum* ssp. *andigena (violet)*	8.91	64.43	80.18		
*S. tuberosum* ssp. *Andig*	traces	11.12	572.20		
Rote Emma	5.12	10.12	15.33	HPLC	[47]
Rosemarie	7.17	12.20	19.37		
Blaue Annelise	8.79	15.66	24.45		
Blaue St. Galler	6.09	11.96	18.05		
Valfi	24.9	58.2	83.1	HPLC	[46]
Blaue Elise	21.2	45.5	66.6		
Red potato	57.2	160.4	-	n.d.	[49]
Gold potato	63.6	130.1	-		
Organic Russet	26.8	59.3	-		
Non-organic Russet	34.7	78.1	-		

* expressed as fresh weight.

**Table 3:** Phenolic compounds and antioxidant activities obtained in different potato peel varieties with different types and conditions of extraction.

Potato Variety	Extraction Conditions	Phenolic Compounds			Antioxidant Activity			Ref
		1	2	3	I	II	III	
**Conventional Extraction**								
Ratona Morada	70% methanol or acetone (*v*/*v*), room temperature, 15 min, 5% (*p*/*v*)	6.29–6.50	-	-	-	-	-	[32]
Curiquinga		8.03–8.78	-	-	-	-	-	
Lady Rosetta	80% methanol, 23 °C, 15 h	3.28	237.36	85.08	3.51	6.27	-	[73]
Lady Claire		2.17	2.16	68.19	1.75	3.45	-	
n.d.	50% methanol, 1:20 S-L, 25 °C, 60 min	6.26	-	-	-	-	-	[74]
Lady Claire	Methanol and 75% ethanol, 80 °C, 22 min	1.26–3.94	-	-	2.00	3.52	-	[34]
**Ultrasound-Assited Extraction (UAE)**								
Ratona Morada	70% acetone, 5% (*p/v*); 50 min, 50 °C	17.70	380.0	-	39.81 *	-	0.410	[32]
Curiquinga		19.10	440.0	-	49.98 *	-	0.412	
Lady Rosetta	80% methanol, 1:10 (*w/v*), 30–45 °C, 30–900 min, 42 and 33 kHz	7.67	267.4	129.05	5.86	22.21	-	[73]
Lady Claire		3.8–4.24	5.98–8.69	118.28–120.83	3.16–3.86	5.64–5.85	-	
**Indirect Ultrasound-Assited Extraction (IUAE)**								
n.d.	50% Methanol, 1:20 S-L, 25 °C, 30 min	9.09	-	-	-	-	-	[74]
**Direct Ultrasound-Assited Extraction (DUAE)**								
n.d.	50% Methanol, 1:20 S-L, 25 °C, 30 min	9.33	-	-	54.2 **	-	-	[74]
**Microwave-Assited Extraction (MAE)**								
Russett Burbank	67.33% Methanol, 15 min, 1:20 S-L, 14.67% level power and room temperature	3.94	-	-	74 **	-	-	[75]
**Sequential Hydrothermal Extraction (SeqHTE)**								
Mixture	water stage 1: 170 °C, 1 MPa, 20 min	17.01 ***	-	-	-	-	-	[63]
	water stage 2: 220 °C, 2.5 MPa, 20 min	3.72 ***	-	-	-	-	-	
**Pressurized Liquid Extraction (PLE)**								
Lady Claire	70% ethanol and 125 °C	3.68	-	-	3.39	-	-	[34]

GAE: Gallic acid equivalent, db: dry biomass, TE: trolox equivalent.; 1. TPC (mg GAE/g db), 2. chlorogenic acid (µg/g db), 3. caffeic acid (µg/g db); I. DPPH (mg TE/g db), II. FRAP (mg TE/g db), III. ABTS+ (mM TE/g db); * EAx10-3, ** %, *** (kg/h).
